# Resuscitative endovascular balloon occlusion of the aorta for ruptured pancreaticoduodenal artery aneurysm

**DOI:** 10.1002/ccr3.3618

**Published:** 2020-12-04

**Authors:** Yuki Mochida, Yasuhiko Miyakuni, Yasuhiko Kaita, Yoshihiro Yamaguchi

**Affiliations:** ^1^ Department of Trauma and Critical Care Medicine Kyorin University School of Medicine Tokyo Japan

**Keywords:** angiography, balloon occlusion, hemorrhagic shock, resuscitation

## Abstract

Resuscitative endovascular balloon occlusion of the aorta is useful as a troubleshooting response to hemorrhage and a temporary method for maintaining patient hemodynamics.

## Graphical abstract

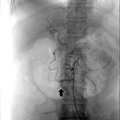

## 1 INTRODUCTION

Pancreaticoduodenal artery (PDA) aneurysms account for about 2% of all splanchnic aneurysms.^1^ They mainly result from hemodynamic changes caused by celiac axis stenosis due to median arcuate ligament compression.^1^, ^2^, ^3^ There are previously published reports of PDA aneurysm with two main lines of treatments being used: endovascular therapy and open repair.^2^, ^3^ Mortality after aneurysm repair is much higher for open repair than for endovascular therapy.^4^ Endovascular therapy is also less invasive and can be performed without major complications.^5^ Therefore, previous reports recommended endovascular therapy as the initial approach.^2^, ^3^, ^4^, ^6^ However, it is necessary to switch to surgical treatment when endovascular therapy fails, as in this case. The longer time period required to stop bleeding while attempting endovascular treatment corresponds to increased hemodynamic instability, and, therefore, surgical treatment becomes more difficult. Therefore, resuscitative endovascular balloon occlusion of the aorta (REBOA) is an important and useful intervention for these cases.

## 2 CASE REPORT

**FIGURE 1:**
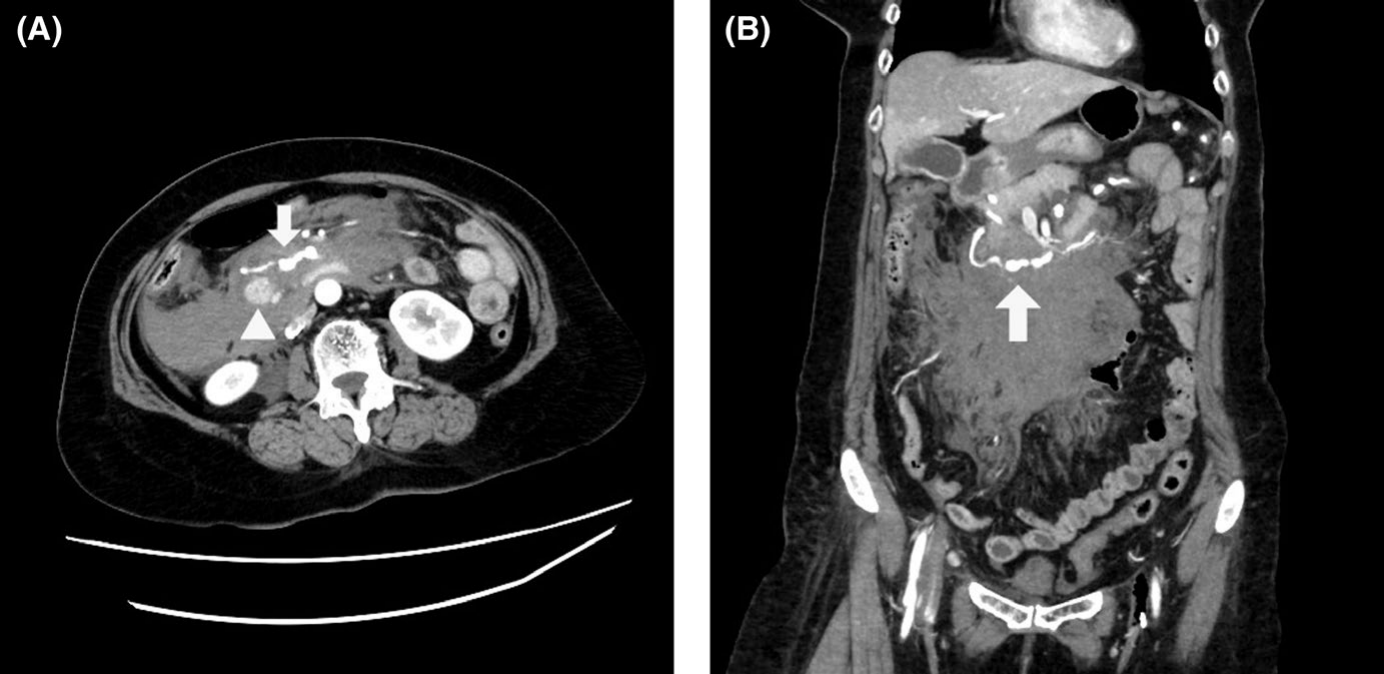
A, Enhanced CT scan showed ruptured PDA aneurysm (arrow) and extravasation (arrowhead). B, Enhanced CT scan (coronal view) showed ruptured PDA aneurysm (arrow) and massive retroperitoneal hematoma

A 61‐year‐old woman with a history of dyslipidemia presented in the emergency room with recurrent vomiting. Her blood pressure was 62/37 mm Hg on arrival, and she was admitted to our critical care center. She had only mild abdominal pain despite abdominal distension. Her face was pale, and there was weak palpation of the radial artery. The patient's hemoglobin level at admission was 12.4 g/dL. A venous sheath was inserted in the left femoral vein, and rapid infusion by crystalloid fluid of approximately 1000 mL was initiated in the resuscitation room. Enhanced computed tomography (CT) scan was performed after confirming increased blood pressure; this revealed a ruptured PDA aneurysm and celiac axis stenosis that was compressed by the median arcuate ligament (Figure 1). We decided to perform transarterial embolization (TAE) as an endovascular treatment. The patient was anesthetized and underwent oral intubation followed by transfer to the emergency angiography room.

The patient's blood pressure suddenly dropped, and she developed hemodynamic shock. Her hemoglobin level dropped to 7.3 g/dL. It was difficult to secure the right femoral artery because the patient was obese. The pulse was weak, and the artery had collapsed as a result of the hemorrhagic shock. After several times to puncture the artery, she developed a subcutaneous hematoma and local arterial dissection was suspected. We attempted to access the left femoral artery; however, this too was difficult to secure. The blood pressure declined gradually, and prompt management to control the hemodynamics became necessary. We decided that the treatment was difficult with interventional radiology and it was necessary to switch to surgical treatment. We also believed that performing REBOA promptly would be more important to maintain the hemodynamics until a surgical intervention can be performed. Surgical exposure of the left femoral artery was performed to insert an aortic balloon occlusion catheter.

**FIGURE 2:**
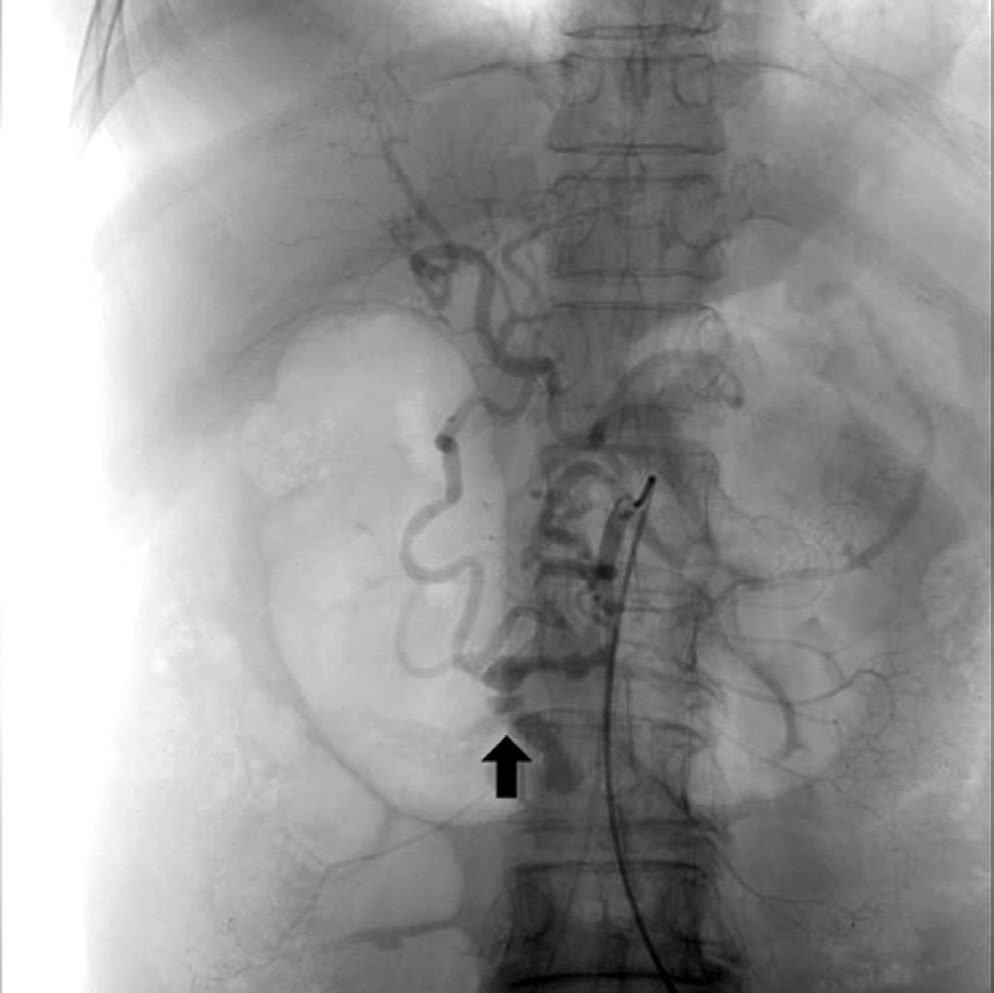
Celiac artery angiography revealing massive extravasation from PDA aneurysm (arrow)

Celiac artery arteriography was performed to confirm the point of rupture before inserting the balloon catheter (Figure 2). The position of the aortic balloon was confirmed using fluoroscopy, and inflation of the balloon resulted in increased blood pressure. Hemodynamics were stabilized, and the patient was moved to an operating room.

Laparotomy revealed a massive retroperitoneal hematoma surrounding the duodenum. Intraoperative bleeding was controlled by REBOA; therefore, the bleeding did not hinder the surgery. The point of bleeding was determined to be the point of rupture that was revealed by collapsing the balloon. The rupture point was confirmed, and both ends of the aneurysm were secured and ligated.

Although there were other visceral aneurysms visualized on the CT scan, we decided against performing any additional interventions since saving life was the priority.

Hemoglobin levels decreased from 12.4 g/dL to 5.5 g/dL. She required 8400 mL of red cell concentrate, 7920 mL of fresh frozen plasma, and 600 mL of platelet concentrate. After intensive care was performed, the patient was extubated on day 8.

A second surgery for incision of the median arcuate ligament and removal of the remaining aneurysm was performed as an elective surgery on day 12.

Finally, the patient was discharged on day 22. She was followed up at the hospital for 6 months. No complications were observed, and no repeat interventions were required.

## 3 DISCUSSION

This case report indicates that REBOA can be a novel option for the treatment of PDA aneurysm. REBOA is useful for hemorrhagic shock and can be easily performed because a low‐profile REBOA device has been developed.^7^, ^8^, ^9^ Recent reports also suggest that REBOA can be performed under ultrasound guidance without fluoroscopy and transferring the patient to an angiography room is not necessary.^10^, ^11^ Therefore, REBOA can be performed promptly and might be able to maintain adequate hemodynamics to the greatest extent possible. Transporting patients with life‐threatening conditions is very dangerous for a facility that has no hybrid operating rooms. In such cases, REBOA is an important intervention to maintain adequate vital signs.

Resuscitative endovascular balloon occlusion of the aorta is also useful for surgery because it can reduce bleeding in the surgical field.^12^ Furthermore, intentional deflation of the balloon during surgery can reveal the rupture point(s). Although orientation in the surgical field is difficult in the context of massive retroperitoneal hematoma, rupture points can be identified by finding the spot from which the blood first emerges.

It should be noted that inflation of the balloon for more than 30 min when performing REBOA should be avoided. An occlusion time of more than 60 min may lead to increasing complications related to ischemia and reperfusion.^13^

Endovascular treatments have been recently used in hemorrhagic diseases. However, since most interventions depend on the endovascular technique, surgical techniques might become redundant over time. Nevertheless, conversion from endovascular to surgical treatment should be undertaken when appropriate.

## 4 CONCLUSION

Although endovascular treatment for hemorrhagic disease is useful, a surgical approach might be necessary if the endovascular approach fails. REBOA is also useful as a troubleshooting response to hemorrhage and might make it easier to perform the surgery.

## CONFLICT OF INTEREST

None declared.

## AUTHOR CONTRIBUTIONS

YM: drafted and wrote the manuscript, and performed surgery; YM and YK: collected data and performed surgery; YY: oversaw manuscript and publishing; all authors read, revised, and approved the final manuscript.

## ETHICAL APPROVAL

Written informed consent was obtained from the patient for publication of this case report and accompanying images.

## Data Availability

All data generated or analyzed during this study are included in this published article.
